# PiProteline: An R Package for Integrated Proteomics Data Analysis, from Label-Free Quantitation to Systems Biology

**DOI:** 10.3390/biology15191723

**Published:** 2026-10-01

**Authors:** Andrea Lomagno, Saleh Hamed, Ishak Yusuf, Pietro Luigi Mauri, Dario Di Silvestre

**Affiliations:** 1 Department of Biomedical Science, Institute for Biomedical Technologies, National Research Council, F.lli Cervi 93, Segrate, 20054 Milan, Italy; lomagno.andrea@hsr.it (A.L.); pierluigi.mauri@itb.cnr.it (P.L.M.); 2 Institute of Experimental Endocrinology and Oncology “G. Salvatore”, National Research Council, Pietro Castellino 111, 80131 Naples, Italy

**Keywords:** proteomics, bioinformatics, systems biology, R package

## Abstract

The demand for user-friendly applications to support biologists in analyzing high-throughput proteomics data remains a pressing challenge. Given the complexity and the multiple intermediate steps involved, this process is time-consuming and often requires specialized computational skills. To simplify and accelerate the exploration of proteomics data, we present PiProteline, an R package designed to operate on high-dimensional data matrices assembled from the output of any search engine commonly used in bottom-up proteomics experiments. In addition to data preprocessing and descriptive statistics, PiProteline enables label-free quantitation, functional enrichment, and systems biology analyses. It supports both unweighted and weighted protein–protein interaction (PPI) network topological analyses for the identification of critical nodes, such as hubs and bottlenecks. By integrating multiple computational approaches, PiProteline accelerates the selection of potentially relevant protein signatures, providing insights into the molecular mechanisms characterizing the investigated systems. This information facilitates the formulation of new hypotheses and the design of targeted experiments, ultimately reducing costs and advancing translational medicine. PiProteline is available as an open-source R package via GitHub, as a Shiny application, and via Zenodo.

## Simple Summary

Proteins are the molecular machines that carry out most functions in our cells, and understanding which proteins change in disease can reveal new ways to diagnose and treat patients. Modern mass spectrometry allows scientists to measure thousands of proteins in a single sample, but turning this raw data into meaningful biological insights requires many complex computational steps, often demanding skills that most biologists do not have. We present PiProteline, a free and open-source software tool that simplifies this process. PiProteline takes protein data produced by any common analysis software and automatically performs quality checks, identifies proteins that differ between healthy and diseased samples, and reveals which biological processes are affected. Uniquely, it also builds networks of protein interactions and pinpoints the most important proteins within them, called hubs and bottlenecks. By combining these different types of analysis, PiProteline helps researchers quickly identify the most promising proteins for further study. The tool is available both as an R package for experienced users and as a user-friendly application for those without programming skills. By making proteomics analysis faster, more standardized, and accessible, PiProteline can help accelerate biomedical discovery and reduce the cost of developing new treatments.

## 1. Introduction

The study of biological systems through multiomic profiling has become an increasingly prevalent approach in modern biology [1]. This strategy serves a dual purpose: it aims to elucidate the molecular mechanisms underlying the phenotypes and genotypes under investigation, while also functioning as a preliminary screening tool for the discovery of potential biomarkers or key regulatory molecules. Since proteins are the primary effectors of most biological functions, their comprehensive investigation is essential to achieving these goals and, consequently, to advancing precision medicine [2]. The foundation for this pursuit was laid by whole-genome sequencing, which catalyzed the development of high-throughput proteomic technologies based on mass spectrometry (MS) and liquid chromatography (LC)—milestones that have profoundly shaped the field [3]. Consequently, it is now possible to analyze the protein composition of complex biological samples—including cell lines and tissues—with remarkable speed, accuracy, and reproducibility, while maintaining high sensitivity and relatively low cost. Furthermore, recent methodological innovations, such as Data-Independent Acquisition (DIA), have enabled increasingly deep proteomic coverage, allowing the qualitative and quantitative characterization of several thousand proteins per sample or analytical run [4].

In addition to the analytical phase, a typical proteomics pipeline workflow comprises several computational steps, including raw spectra interpretation, data preprocessing, protein quantitation, and functional evaluation [5]. Regarding raw spectra interpretation, both commercial and free-of-charge software are available [6]. Many of these tools are already integrated with utilities that provide a comprehensive package of results, including protein profiles, differentially expressed proteins, and enriched Gene Ontology (GO) terms and biological pathways. However, the variety of MS search engine algorithms, formats, statistics, and data filtering approaches makes post-protein identification data analysis challenging. Consequently, the evaluation of the same raw data through different computational pipelines can yield divergent results [7].

To simplify proteomics data exploration across multiple platforms and create tools that can be assembled into logical, customizable, and reproducible workflows, several R packages addressing specific needs have been released, including those for protein quantitation [8,9,10], statistical analysis [11], and functional assessment of multiomics data [12]. Packages that integrate all these functions, providing a more comprehensive set of results, have also been proposed [13,14].

Although the scientific community is actively addressing these challenges, the need for easy-to-use applications to support biologists in synthesizing proteomic data remains current. Indeed, this workflow, which involves multiple analysis steps, is time-consuming and often requires specific IT skills. To facilitate these activities, we present PiProteline. Developed as an R package and Shiny application, it operates on high-dimensional data matrices assembled from the output of any search engine software commonly used in bottom-up proteomics experiments. In addition to data preprocessing, such as cleaning and normalization, PiProteline enables descriptive statistics, differential expression analysis, and functional enrichment, accompanied by result visualization. Notably, it also supports both weighted and unweighted network analyses with automated selection of the corresponding hubs and bottlenecks [15,16].

## 2. Materials and Methods

### 2.1. PiProteline R Code and Shiny APP

PiProteline runs on all major operating systems, and the released version is available via GitHub as an open-source R package (https://github.com/lomi95/PiProteline, accessed on 1 September 2026);the data used for testing PiProteline, and the corresponding outputs, including tables and graphs, are available. Full documentation is provided within the README.md files. For PiProteline R package development, key libraries include *tidyverse* for data manipulation and visualization [17], *rbioapi* for functional enrichment [18], and *igraph v2.1.4* for graph analysis [19].

A Shiny web application (https://github.com/salehnhd/PiProteline-Shiny, accessed on 1 September 2026) integrating PiProteline’s functionality was developed using RStudio v2025.9.0.387. The interface was built using *shiny v1.11.1* [20], *shinyWidgets v0.9.0* [21], *DT v0.34.0* [22], and *ggplot2 v3.5.2* [23]. PiProteline is also available via Zenodo (https://doi.org/10.5281/zenodo.22732169).

### 2.2. Data Test

To test PiProteline’s functionality, we used a set of proteomic profiles obtained from the analysis of substantia nigra samples from healthy controls (C, *n* = 20), patients with Parkinson’s disease (PD, *n* = 20), and patients with Parkinson’s disease carrying a GBA1 mutation [24]. Specifically, spectral counts (SpCs) per identified protein were used as the quantitative parameter. Moreover, to validate the reliability of PiProteline in the context of quantitative analsyis, we processed a public dataset (PRIDE ID: PXD001819) containing a known set of 48 human proteins spiked onto a fixed yeast protein background, at nine different known concentrations [25].

## 3. Results

### 3.1. PiProteline Workflow

PiProteline supports the output of any search engine software commonly used in bottom-up proteomics experiments, including MaxQuant, Proteome Discoverer, Spectrum Mill, and Skyline [6]. It works with data obtained from both data-dependent acquisition (DDA) and data-independent acquisition (DIA), adequately formatted as a high-dimensional data matrix *m × n* (Figure 1). The main PiProteline steps include data pre-processing, label-free differential expression, functional enrichment and network analysis (Figure 2). Compared to other similar R packages, network analysis represents a novel feature. In particular, it enables both unweighted and weighted network topological analyses with automated selection of critical nodes, such as hubs and bottlenecks [15], which are combined with differential expression analysis for the selection of the most putatively relevant proteins [16].

#### 3.1.1. Pre-Processing

Although specific computational tools have recently been developed to address this issue [26,27], bottom-up proteomics still suffers from inherent limitations that frequently complicate the accurate quantification of protein isoforms [28]. In a typical proteomics data matrix generated by searching MS raw spectra against a FASTA database, multiple protein isoforms mapping to the same gene name may be present. PiProteline resolves this redundancies by retaining by retaining the maximum value (AU, i.e., SpC/Intensity/Peak Area) per sample as illustrated in (Figure 3A). While recent versions of search engines such as Proteome Discoverer and MaxQuant automatically mitigate such redundancies, we acknowledge that our approach may impose constraints on the precise quantification of individual isoforms. Nevertheless, because the resulting protein abundance profiles are intended for network-based analyses, our primary goal is to construct protein–protein interaction (PPI) network models that can be examined at both functional and topological levels. Importantly, PPI network reconstruction via STRING does not discriminate among isoforms but instead relies on the gene name as the primary identifier [29], thus justifying our aggregation strategy in this specific analytical context. network models it is equally necessary to emphasise that, our strategy is justified by the fact that the construction of protein–protein interaction, since we refer to the gene name as the primary key for building protein–protein interaction (PPI) network models.

**Figure 1:**
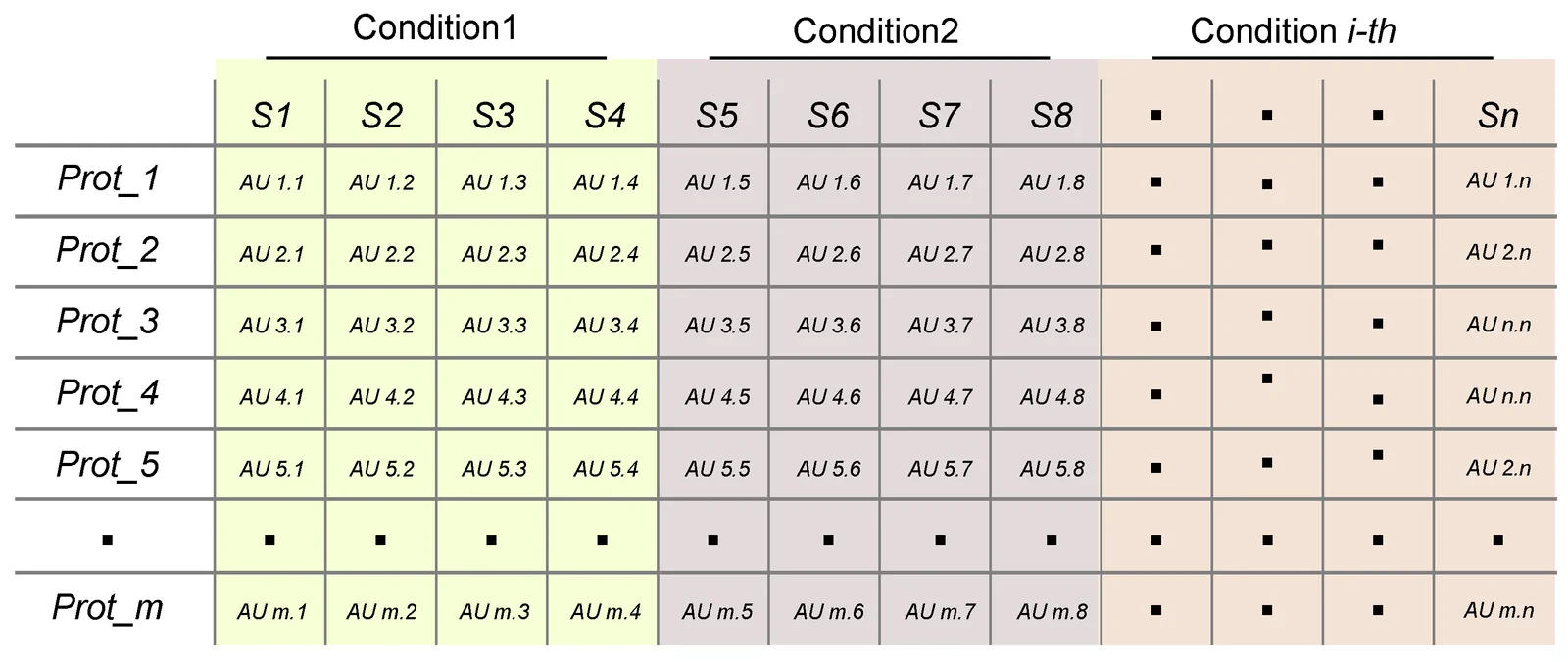
**Overview of PiProteline input data structure.** PiProteline accepts high-dimensional data matrices formatted as *m* × *n* where, rows *m* represent proteins and columns *n* represent individual samples. The input can be derived from the output of any search engine software commonly used in bottom-up proteomics experiments (e.g., MaxQuant, Proteome Discoverer, Spectrum Mill, Skyline) and supports data obtained from both data-dependent acquisition (DDA) and data-independent acquisition (DIA). The matrix contains quantitative values expressed as arbitrary units (AUs), such as spectral counts (SpCs), peak area, or intensity, depending on the quantification method employed [30].

**Figure 2:**
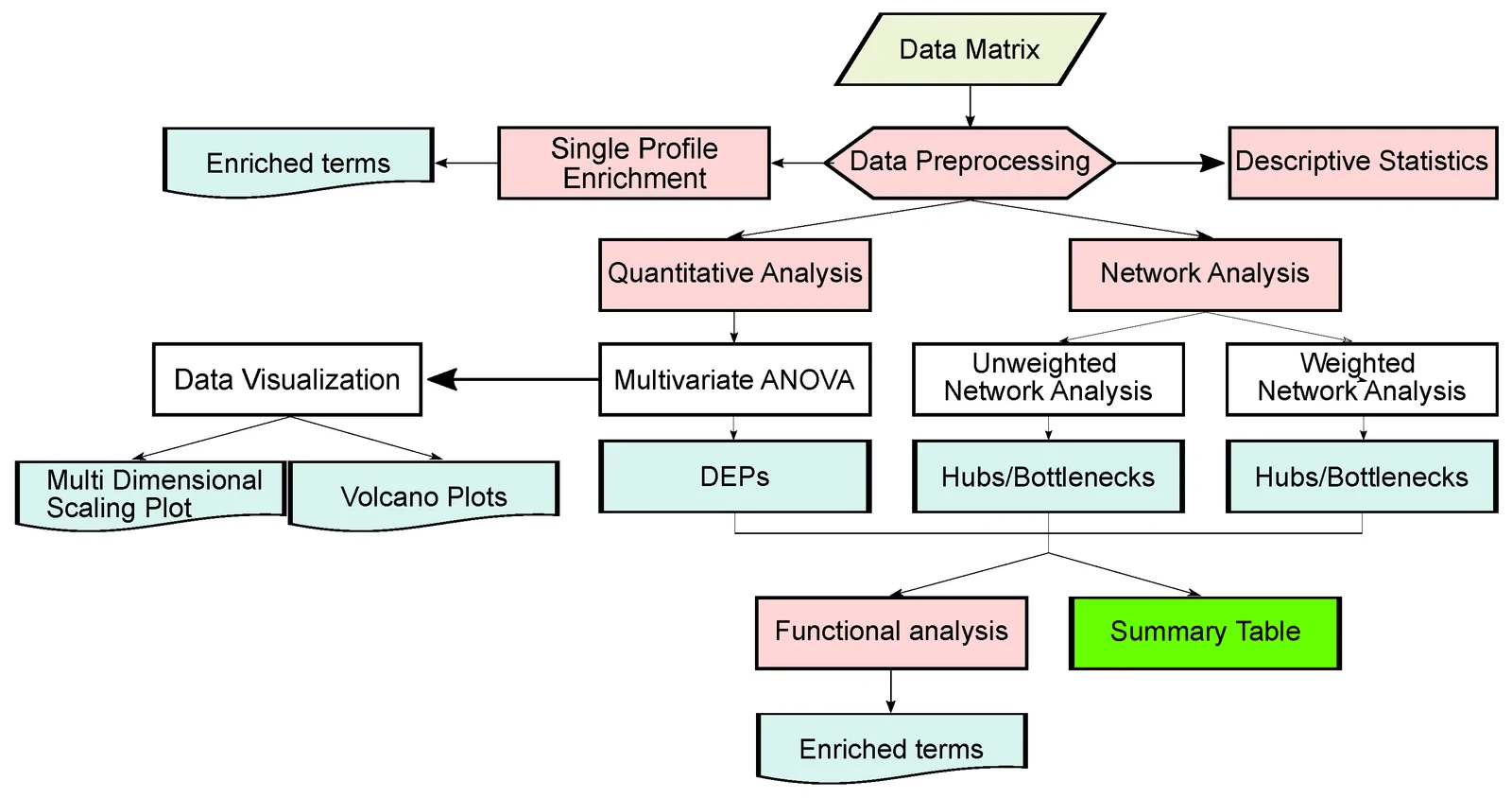
**PiProteline workflow flowchart.** Diagram summarizing the main steps of PiProteline R package. These include data preprocessing, descriptive statistics, quantitative analysis, network analysis, and functional enrichment analysis. Differentially expressed proteins (DEPs) and topologically relevant nodes, such as hubs and bottlenecks, represent intermediate outputs that feed into subsequent analytical steps; specifically, the pink and white blocks indicate the methodological approaches, while the blue ones indicate the results obtained from them.

**Figure 3:**
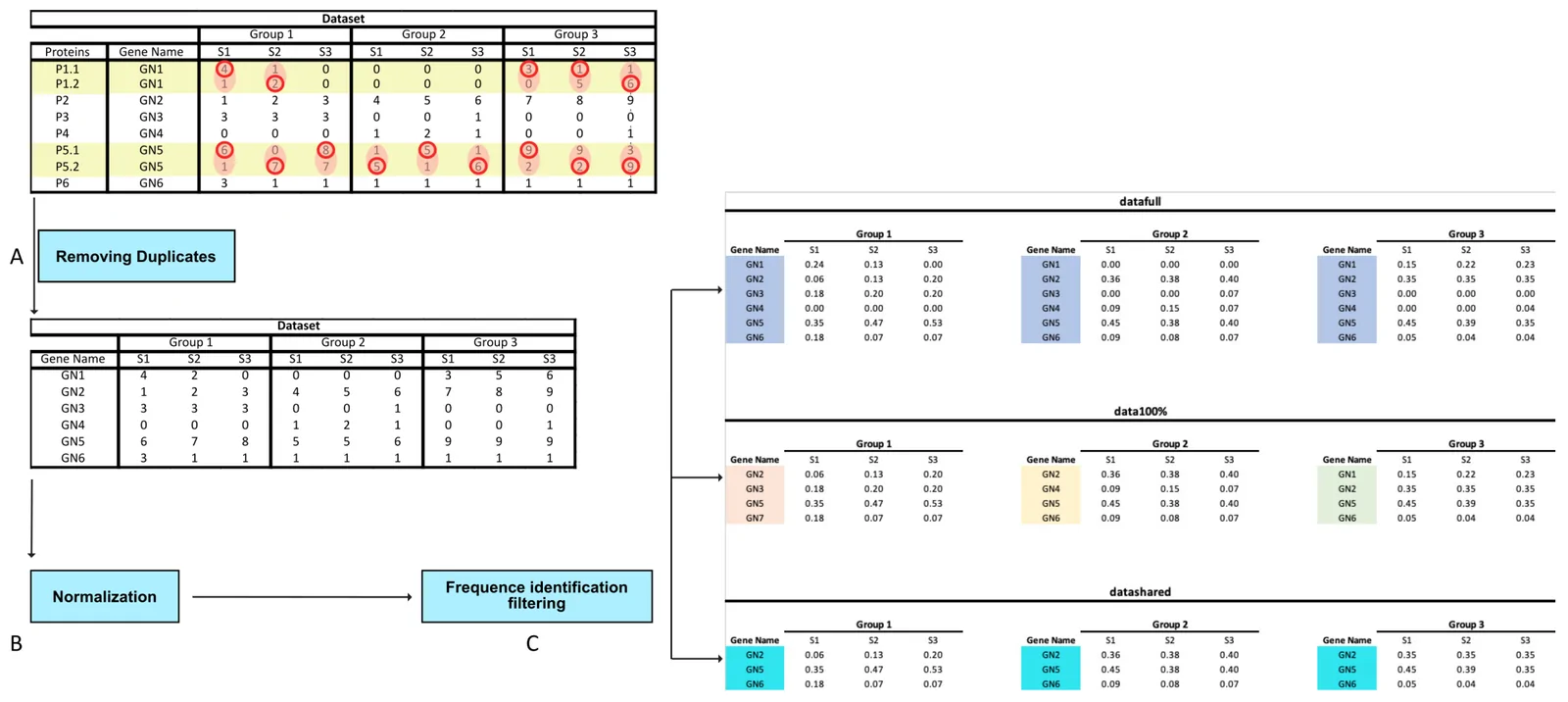
**Data preprocessing workflow.** (A) The original data matrix is processed to remove duplicate entries corresponding to protein isoforms by retaining the maximum abundance value per sample. (B) Data normalization is performed using various strategies; total signal normalization is shown as an example, which transforms the data so that each sample (column) exhibits an equal sum of spectral counts (SpCs), intensity, or peak area. (C) Three intermediate datasets, *datafull*, *data100%* and *datashared*, are generated based on identification frequency (IF) thresholds. *datafull* contains all identified proteins; *data100%* includes only proteins found in all samples within each group/condition (IF = 100% per group); and *datashared* comprises proteins identified in all samples across all experimental conditions (IF = 100% across all groups).

After removing redundancies, the data may be normalized using different strategies, including total signal normalization, Z-score normalization, maximum signal normalization, row sigma normalization, unit vector normalization, min-max normalization, and logarithmic transformation [31] (Figure 3B). Finally, the data are filtered based on protein identification frequency (IF).

A first dataset, referred to as *datafull*, contains all proteins. A second dataset, referred to as *data100%*, extracts for each group/condition only proteins found in all samples (IF = 100% per group/condition). PiProteline then generates separate group-specific lists for Unweighted Network Analysis (*data_grouped_una*) and Weighted Network Analysis (*data_grouped_wna*). Their minimum within-group IF thresholds (unweighted_if and weighted_if, respectively) are user-adjustable and both default to 100%, thus retaining proteins identified in every sample of a group/condition. A complete cross-group list, termed data_grouped_shared (*datashared*), is also created by considering only proteins found in all samples across all conditions (IF = 100% in all groups/conditions). Both *data_grouped_wna* and *datashared* can be used as input for the WNA; in the case of *data_grouped_wna*, the set of proteins considered for each group may differ because it accounts for intra-group IF, whereas with *datashared*, each group is evaluated against the same set of proteins, and the distinguishing factor is solely the level of correlation between the proteins within the different groups. For convenience, we refer to lists of datasets rather than individual datasets, since filters are applied according to the studied conditions or groups (Figure 3C).

Before proceeding to the next steps, PiProteline performs exploratory data analysis to assess missing values, as well as the mean, minimum, and maximum protein abundances within the dataset, and the mean and standard deviation values for individual samples. Moreover, *Spearman*’s correlation between groups is calculated using the entire protein dataset.

#### 3.1.2. Quantitative Analysis

PiProteline’s protein quantitation is based on a label-free strategy that exploits spectral counts (SpCs), peak area, or intensity as quantitative parameters. When the data matrix contains more than six replicates per condition, multivariate ANOVA (MANOVA) and Benjamini–Hochberg correction are applied [32]; by the term “replicates”, we mean biological replicates, or at least three biological replicates, each analyzed in two technical replicates. MANOVA also works with a number of replicates less than six (and greater than two), providing a warning. However, since PiProteline is designed for label-free quantification, we discourage following this procedure unless for purely exploratory purposes, followed by an independent validation process [33].

Differentially expressed proteins (DEPs) are extracted by processing the complete normalized data matrix (*datafull*), which is used both for comparing all groups simultaneously (when more than two groups/conditions are available) and in pairwise comparisons. In any case, fold change and Differential Average (DAve) index are calculated to establish the expression trend in pairwise comparisons [5].

Once DEPs are extracted, they serve as input for the Random Forest model [34], which calculates the distance between the groups/conditions analyzed. This distance allows reconstruction of a multidimensional scaling (MDS) plot, reducing the original dataset’s dimensionality and visually displaying the separation between groups/conditions and the amount of variance explained (Figure 4).

The features (DEPs) extracted are also used to build a volcano plot, where the x-axis represents the fold change (FC) and the y-axis displays −$\log_{10}\left(adj.p\text{-}\mathrm{value}\right)$. Both parameters can be filtered by setting appropriate thresholds (e.g., adj.p-value≤0.05 and |FC|≥1) to select DEPs with the desired level of significance (Figure 5).

Furthermore, protein expression variation in pairwise comparisons is evaluated through the DAve index, which is incorporated into the output files alongside other statistical parameters.

Specifically, fold change (FC) is defined as:

$$\mathrm{FC}=\log_{2}\left(\frac{AU_{X}+\kappa }{AU_{Y}+\kappa }\right)$$

where κ is a default pseudocount (κ=0.01)

Meanwhile, the Differential Average (DAve) index is defined as:

$$\mathrm{DAve}=\frac{\left(AU_{X}-AU_{Y}\right)}{\left(AU_{X}+AU_{Y}\right)}\times 2$$

where $AU_{X}$ and $AU_{Y}$ represent the quantitative parameter (expressed as arbitrary unit, AU, i.e., spectral count, peak area or intensity) of a given protein in two compared groups/conditions (X and Y). By convention, the DAve sign (+/−) indicates the expression trend, with positive DAve denoting proteins up-regulated in the first group/condition (X) and negative DAve denoting proteins up-regulated in the second group/condition (Y) [5].

To validate the reliability of PiProteline in the context of quantitative analysis, we measured its performance against other similar tools, which include DEP [35], protti [14], tidyproteomics [13], and limma [36,37]. To achieve this goal, we processed a dataset containing a fixed yeast protein background, and a set of 48 human proteins at nine different known concentrations [25]. Overall, PiProteline provided results in line with those of widely used and published tools. While not the best (Limma showed the best performance), out of six tools tested, PiProteline ranked third, with a performance range comparable to that of DEP and protti (see Supplementary Information, Figure S1).

### 3.2. Network Analysis

PiProteline enables network topological analysis through two different strategies: Unweighted Network Analysis (UNA) and Weighted Network Analysis (WNA). However, to obtain reliable results, it is not recommended to rely on WNA if fewer than 20–30 samples/profiles per group/condition are available [38]. UNA uses *data_grouped_una*, which is filtered within each group/condition using the user-adjustable unweighted_if threshold. WNA uses *data_grouped_wna*, which is filtered within each group/condition using the independently user-adjustable weighted_if threshold. Both defaults are 100%; the two analyses therefore use analogous IF filtering while retaining separate inputs and settings. In WNA, the PPI graph is weighted through modified *Spearman*’s correlation calculated from the corresponding *data_grouped_wna* matrix; optionally, the more conservative *datashared* matrix can be used for analyses that require a common protein universe across groups. When weighted_if is lower than 100%, non-detections are treated as missing values and correlations are calculated from pairwise-complete observations. The selected IF threshold and missing-value/correlation policy are returned with the analysis results and should be reported. PiProteline warns users when WNA is attempted with fewer than 20 samples per group; results from smaller datasets should be considered exploratory.

For both UNA and WNA, protein–protein interactions (PPIs) models are reconstructed starting from STRING-db [39]. For UNA, an undirected and unweighted graph is reconstructed, while for WNA, an undirected graph is generated and subsequently weighted using a modified *Spearman*’s correlation score [16] calculated from the experimental data matrix (*datashared*).

After PPI graph reconstruction, the largest connected component is extracted and analyzed topologically. Disconnected or isolated nodes are removed. Centrality measures, including betweenness, centroid, and bridging, are calculated to identify critical nodes, such as hubs and bottlenecks [15,16]. Specifically, they are defined as:

- Hubs: Nodes with betweenness and centroid values greater than the 75th percentile.
- Bottlenecks: Nodes with betweenness and bridging values greater than the 75th percentile.

Hubs and bottlenecks are further classified into three categories (Table 1):

- NotSpecific: Nodes whose centralities meet the 75th percentile criteria in any group/condition.
- Specific: Nodes whose centralities meet the 75th percentile criteria in a group/condition but not in all others.
- Centrality Specific: Nodes whose centralities satisfy the 75th percentile criterion in one group/condition, while in all others no centralities exceed the threshold.

### 3.3. Functional Analysis

Through the STRING API, PIProteline programmatically queries the STRING database to extract the most enriched GO terms and Pathways [29]. Functional enrichment analysis was performed by standard ORA (Over Representation Analysis) on Gene Ontology (GO), Reactome, KEGG, and WikiPathways databases as references. A single-profile enrichment considers the proteins found in each individual sample. Following the processing of all samples, a *t* × s data matrix is generated, where *t* represents the enriched term and *s* represents the sample in which it was enriched.The generic element $t_{i}s_{j}$ reports the number of proteins in the j-th sample that allowed the enrichment of the i-th term. MANOVA is then applied to select GO terms and pathways that are differentially enriched among the considered groups/conditions (Figure 6A).

Functional enrichment is also performed using DEPs from quantitative analysis, and hubs and bottlenecks from network analysis. For protein quantitation, enrichment is conducted on DEPs extracted from each pairwise comparison. To simplify the output, PiProteline provides a table with exclusive differentially enriched terms (e.g., if a term is enriched from both up- and down-regulated proteins in X *vs.* Y comparison, it is removed from the output) (Figure 6B).

Enrichment analysis is finally carried out using topologically relevant proteins extracted from network analysis. The enrichment is evaluated for both UNA and WNA outputs, as well as for each group/condition under study. As for DEPs, to provide group-specific information, pairwise comparisons are performed and an output table reporting exclusive terms is created.

To integrate quantitative and network analyses, the terms enriched in both steps are compared for each pairwise comparison. A list is generated distinguishing terms with consistent trends (e.g., in the X *vs.* Y comparison, terms identified exclusively in X *vs.* Y) and terms with opposing trend (e.g., terms enriched in X based on quantitative analysis but enriched in Y based on network analysis, or vice versa).

### 3.4. Summary Tables

To summarize the volume of information generated by PiProteline, different output tables are provided. They include:

- **output_unfiltered_results**: Contains all unfiltered results (i.e., Quantitative Analysis (*all vs. all*), Quantitative Analysis (pairwise comparisons), UNA, WNA, Summary).
- **output_significative_results**: Contains all results filtered based on the thresholds set (i.e., Quantitative Analysis (*all vs. all*), Quantitative Analysis (pairwise comparisons), UNA (NotSpecific, Specific, CentralitySpecific), WNA (NotSpecific, Specific, CentralitySpecific).
- **output_enrichment**: Includes single-profile enrichment and enrichment from quantitative and network topology pairwise comparisons.
- **output_enrichmentTrend**: Includes tables reporting GO terms and pathways with the same or opposing trends from quantitative and network analysis.

The Summary table, included in the **output_unfiltered_results** file, encompasses all data processing steps. Notably, it also includes two additional columns:

- **Total Significance Count**: The number of analyses in which a given protein was found to be significant.
- **Weighted Significance Score**: A weighted score normalized based on the type of analysis in which a given protein was found to be significant.

The score is calculated as follows:

(1) Score=(8×M)+(2×w)+(2×s)+(4×cs)

where:

- *M* = number of times a given protein was significant in pairwise quantitative comparisons (MANOVA);
- *w* = number of times a given protein was significant by WNA;
- *s* = number of times a given protein was identified as *specific* critical node across UNA and WNA analyses;
- cs = number of times a given protein was identified as *centrality-specific* critical node across UNA and WNA analyses.

The weights (8, 2, 2, and 4) were empirically selected to give greater emphasis to reproducible significance in quantitative pairwise comparisons (*M*), by assigning a secondary contributions to WNA significance (*w*) and *specific* critical node identification (*s*), and finally by giving additional weight to *centrality-specific* critical nodes (*cs*). By sorting the Summary table by total significance count or weighted significance score, proteins that were found to be significant across multiple analyses can be quickly identified. Thus, this score provides an immediate overview of proteins that potentially represent promising targets for further investigation and targeted experiments.

## 4. Discussion

The PiProteline R package presented here aims to expedite a computational pipeline for comprehensive evaluation of high-throughput proteomics results following protein identification. It offers the advantage of automating complex and repetitive analyses, reducing human error, and improving overall workflow efficiency. In addition to streamlining these steps, PiProteline contributes to increased standardization, facilitating the comparison of results obtained from different studies, as well as from different analytical instruments and search engine software. Indeed, the intrinsic heterogeneity of proteomic workflows, arising from the combination of multiple analytical and computational tools, exacerbates the low reproducibility of results obtained even from the same raw data [7]. In this scenario, PiProteline showed comparable performance to similar tools, such as DEP [35], protti [14], tidyproteomics [13] and limma [36,37]. Its reliability was further confirmed by a preliminary evaluation of the results obtained from processing the test data sets. This is supported by previously established evidence, such as the role of translational machinery alterations in disease pathogenesis and the presence of mitochondrial dysfunction in the substantia nigra (SN) of Parkinson’s disease patients [16,40,41].

Overall, PiProteline is intended as a flexible and integrative tool for exploratory and comparative proteomic analysis. As an increasing number of studies rely on high-throughput proteomic data, and the volume of data produced continues to grow, the availability of tools that simplify and standardize data processing represents a competitive advantage, freeing up valuable time for subsequent investigations and facilitating future clinical applications [42]. For this purpose, we emphasize that, in addition to quantitative and functional analysis, PiProteline is the only tool that integrates weighted and unweighted network topology for the selection of hubs and bottlenecks [15,16]. This information, integrated with traditional quantitative analysis, represents a fundamental step in filtering the most potentially interesting molecules, thereby accelerating the discovery of markers through targeted experiments that reduce unnecessary costs and facilitate translational medicine [43].

PiProteline’s modularity and flexibility allow users to tailor each step to the specific characteristics of their dataset and objectives. In this regard, we note that, since PiProteline operates on a high-dimensional data matrix assembled from the output of any search engine software commonly used in bottom-up proteomics experiments, its potential applications are very broad [44]. Furthermore, the development of a Shiny application aims to facilitate and promote the use of PiProteline even for users without advanced bioinformatics skills and/or those primarily dedicated to other fundamental steps of the proteomic workflow, such as sample preparation or management of the analytical-instrumental phase.

## 5. Conclusions

Although we consider this version of PiProteline to be functional and useful for the proteomics scientific community, we are aware that it can be improved. From the perspective of future developments, the R environment facilitates the integration of new features, such as the implementation of existing steps or the addition of new ones customized for specific data types, including transcriptomics and metabolomics. These improvements could include new strategies for quantitative analysis (both labeled and label-free), the use of signaling networks, as well as pipelines for multiomics data integration [45]. Additionally, functional improvements to the Shiny application, including results visualization and sharing on a web server, will represent further steps towards making PiProteline increasingly easy to use for an even wider segment of the scientific community dedicated to the analysis of omics data.

## Figures and Tables

**Figure 4:**
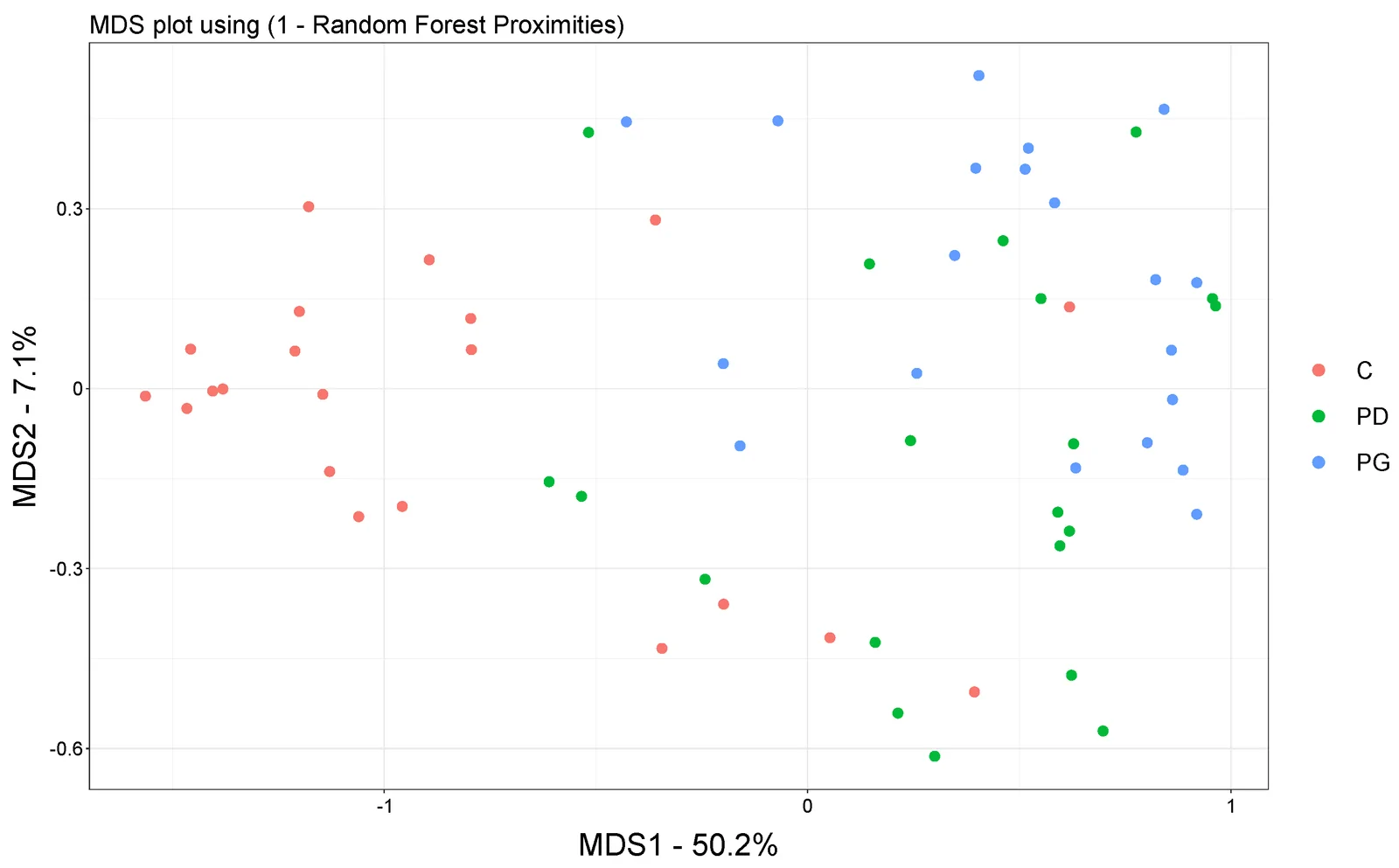
**Multidimensional scaling (MDS) plot.** Using the results of the MANOVA analysis, PiProteline generates an MDS plot to rapidly assess how well the characterized profiles, as well as the differentially expressed proteins, discriminate between the groups/conditions under study (C, PD, PG). Each point represents an individual sample, and distances between points reflect their pairwise dissimilarities. The first two dimensions (MDS1 and MDS2) account for the major variance, clearly highlighting the separation between the experimental groups/conditions.

**Figure 5:**
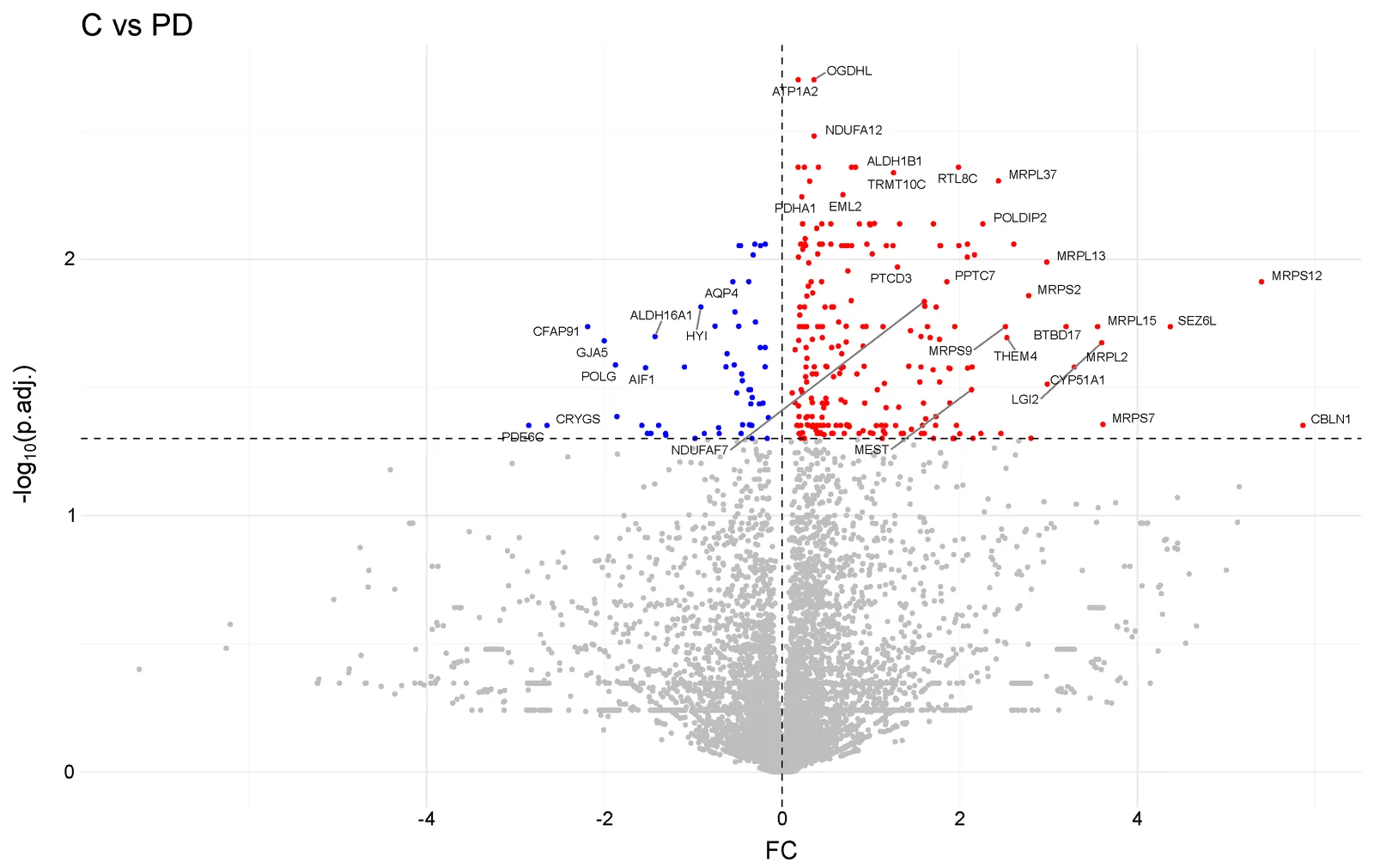
**Volcano Plot.** Representative volcano plot from the comparison between control (C) and Parkinson’s disease (PD) groups. The x-axis represents the fold change (FC), while the y-axis displays the −$\log_{10}\left(adj.p\text{-}\mathrm{value}\right)$. Proteins that pass the significance thresholds (|FC| and *adj.p*-value) are highlighted in red (up-regulated in C) or blue (down-regulated in C), respectively.

**Figure 6:**
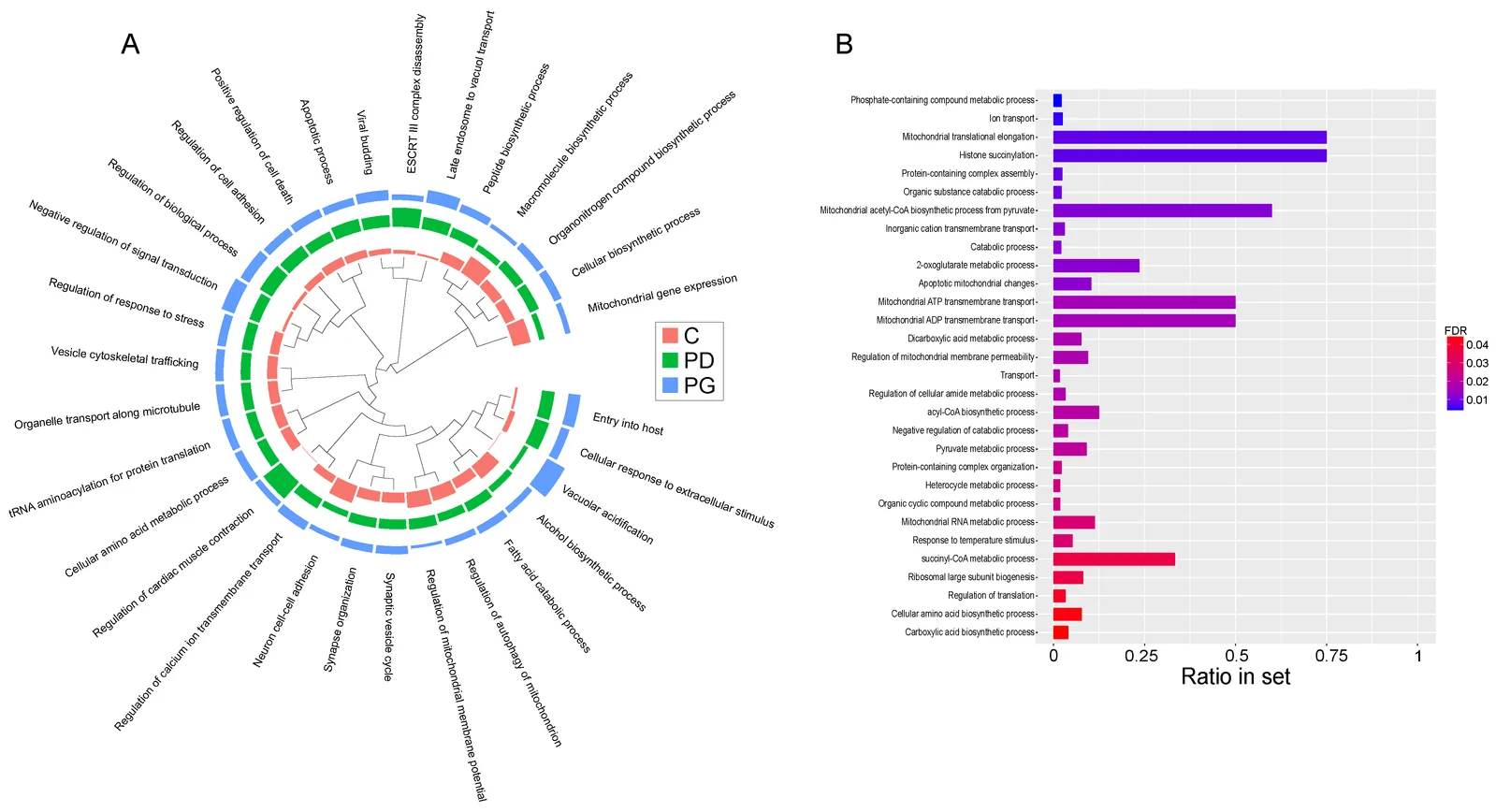
**Functional enrichment analysis.** (**A**) Example of biological processes (BP) enriched using the single-profile enrichment strategy. BP terms are arranged in a tree structure according to the number of shared proteins, while the corresponding bars are proportional to the number of proteins (normalized 0–100) identified for a given BP in each color-coded group/condition. (**B**) Example of a bar plot showing the most enriched BPs (exclusive) in pairwise comparisons (C vs. PD) using DEPs or hubs and bottlenecks. The x-axis reports the ratio of proteins within the set, and bar color indicates the false discovery rate (FDR), where darker blue shades correspond to more significant terms.

**Table 1:** **Classification of hub proteins based on network centrality measures.** The table shows representative threshold patterns for classifying hub proteins in a reference group (G1). Bet and Cen denote Betweenness and Centroid centralities, respectively. A protein is a hub in G1 only when both measures exceed the group-specific 75th-percentile (Q3) threshold. It is classified as *non-specific hub* if it is also a hub in at least one other group. It is classified as *specific hub* if it is hub in G1 but not in both G2 and G3 (i.e., at maximum, in G2 and G3, one centrality exceeds the threshold Q3). It is classified as *centrality-specific hub* if neither centrality exceeds the threshold Q3 in both G2 and G3. A protein that does not meet both criteria is classified as *not a hub* in G1. The symbol “-” indicates that neither measure exceeds Q3. The classification is repeated with each group in turn as the focal group.

Proteins	G1 (Focal)	G2	G3	G1 Hub Type
P1	Bet (>Q3) AND Cen (>Q3)	Bet (>Q3) AND Cen (>Q3)	-	*non-specific hub*
P2	Bet (>Q3) AND Cen (>Q3)	Bet (>Q3)	-	*specific hub*
P3	Bet (>Q3) AND Cen (>Q3)	-	-	*centrality specific hub*
P4	Bet (>Q3)	-	-	*not a hub*

## Data Availability

The data used to generate the results are available (https://github.com/lomi95/PiProteline, accessed on 1 September 2026). The original dataset is publicly available from the study “Large-scale proteomics analysis of five brain regions from Parkinson’s disease patients with a GBA1 mutation” (DOI: https://doi.org/10.1038/s41531-024-00645-x). The PiProteline R package is available on GitHub (https://github.com/lomi95/PiProteline, accessed on 1 September 2026), as well as the corresponding Shiny web application (https://github.com/salehnhd/PiProteline-Shiny, accessed on 1 September 2026).

## Supplementary Materials

The following supporting information can be downloaded at: https://www.mdpi.com/article/10.3390/biology15191723/s1, Figure S1: Comparison of PiProteline and similar tools. (A) Comparisons performed (P1 vs. P4, 10-fold difference; P2 vs. P4, 5-fold difference) starting from a data matrix obtained by processing a public benchmark dataset (PRIDE ID:PXD001819). (B) Sensitivity, FDR and F1 values obtained in P1 vs. P4 and in (C) P2 vs. P4 comparisons. (D) List of features characterizing PiProteline and a set of similar tools already available; DDA/DIA data, Quantification, Differential Expression (DE) statistics, Functional Enrichment and Network Analysis features were considered.

## Abbreviations

The following abbreviations are used in this manuscript:

AU	Arbitrary Unit
BP	Biological Process
C	Control
DAve	Differential Average
DDA	Data-Dependent Acquisition
DEP	Differentially Expressed Protein
DIA	Data-Independent Acquisition
FC	Fold Change
FDR	False Discovery Rate
GO	Gene Ontology
IF	Identification Frequency
KEGG	Kyoto Encyclopedia of Genes and Genomes
LC	Liquid Chromatography
MANOVA	Multivariate Analysis of Variance
MDS	Multidimensional Scaling
MS	Mass Spectrometry
PD	Parkinson’s Disease
PG	Parkinson’s Disease with GBA1 Mutation
PPI	Protein-Protein Interaction
SpC	Spectral Count
UNA	Unweighted Network Analysis
WNA	Weighted Network Analysis
