# Supplementary material for: PiProteline: An R Package for Integrated Proteomics Data Analysis, from Label-Free Quantitation to Systems Biology
Source: Biology (Basel). 2026 Oct 1;15(19):1723. doi: 10.3390/biology15191723 (PMC13663379; doi:10.3390/biology15191723)
Supplement: Supplementary file 1 [file biology-15-01723-s001.zip › Definitions/drawing-eps-converted-to.pdf]

A

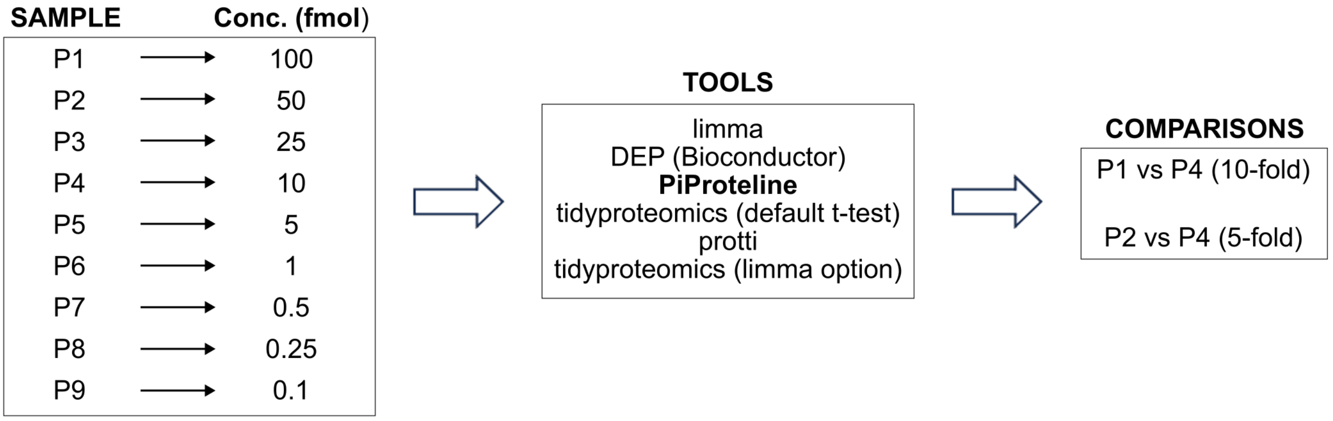

B

Tool	Called	Correct	Wrong	Missed	Sensitivity	FDR	F1
limma	67	46	21	2	0.958	0.313	0.800
DEP (Bioconductor)	70	47	23	1	0.979	0.329	0.797
**PiProteline**	73	46	27	2	0.958	0.370	0.760
tidyproteomics (default t-test)	78	47	31	1	0.979	0.397	0.746
protti	86	47	39	1	0.979	0.453	0.701
tidyproteomics (limma option)	90	47	43	1	0.979	0.478	0.681

C

Tool	Called	Correct	Wrong	Missed	Sensitivity	FDR	F1
tidyproteomics (limma option)	52	46	6	2	0.958	0.115	0.920
limma	50	45	5	3	0.938	0.100	0.918
**PiProteline**	48	43	5	5	0.896	0.104	0.896
protti	55	46	9	2	0.958	0.164	0.893
DEP (Bioconductor)	54	45	9	3	0.938	0.167	0.882
tidyproteomics (default t-test)	1	1	0	47	0.021	0.000	0.041

D

TOOL	DDA/DIA	Quantification	DE statistics	Functional Enrichment	Network Analysis	Other Notable Features
PiProteline	**YES** (output from any search engine)	**YES** (label-free)	MANOVA, Benjamini-Hochberg	**YES**	**YES** (unweighted and weighted network topological analysis for selecting hubs)	R package and Shiny app available on GitHub
aLFQ	**YES** (DDA-LFQ, DIA-LFQ, and plexDDA)	**YES** (IBAQ, TPA values)	Log-Log Linear Regression Monte Carlo Cross-Validation (MCCV)	**NO**	**NO**	Python library (ibaqpy). Scalable for big data
protti	**YES** (output from any search engine)	**YES**	Fixed-Effects Models	**YES**	**YES** (Network analysis using STRING database)	Provides extensive QC functions and direct access to databases like UniProt
mixOmics	**YES** (if already processed)	**NO**	sPCA, sPLS-DA, rCCA, DIABLO, MINT, for dimensionaly reduction	**NO**	**YES** (Relevance Network Graphs, Circos plots for multi-omics)	Designed for data integration and visualization of high-dimensional multi-omics datasets
leapR	**YES** (if already processed)	**NO**	Fisher's exact test, Kolmogorov-Smirnov test, Student's T-Test	**YES**	**YES** (specific for Kinases)	Can calculate enrichment in specified interactions relative to non-pathway members
tidyproteomics	**YES** (data from MaxQuant, PD , FragPipe, Skyline, DIA-NN)	**YES** (label-free, TMT)	Empirical Bayes Student's T-Test	**YES**	**NO**	Focuses on creating a tidy data object for flexible and standardized analysis; supports common data platforms and formats
promor	**YES**	**YES** (label-free)	Linear models and Empirical Bayes	**NO**	**NO**	A comprehensive R package for label-free proteomics data analysis
DEP (Bioconductor)	**YES**	**YES** (label-free, TMT)	Empirical Bayes, Benjamini-Hochberg	**YES**	**NO**	
limma	**YES** (output from any search engine)	**YES** (label-free)	Empirical Bayes	**NO**	**NO**	Often used in proteomics workflows for differential analysis
