# Supplementary material for: PiProteline: An R Package for Integrated Proteomics Data Analysis, from Label-Free Quantitation to Systems Biology
Source: Biology (Basel). 2026 Oct 1;15(19):1723. doi: 10.3390/biology15191723 (PMC13663379; doi:10.3390/biology15191723)
Supplement: Supplementary file 1 [file biology-15-01723-s001.zip › Definitions/logo-mdpi-eps-converted-to.pdf]

MDPI
