# Supplementary material for: PiProteline: An R Package for Integrated Proteomics Data Analysis, from Label-Free Quantitation to Systems Biology
Source: Biology (Basel). 2026 Oct 1;15(19):1723. doi: 10.3390/biology15191723 (PMC13663379; doi:10.3390/biology15191723)
Supplement: Supplementary file 1 [file biology-15-01723-s001.zip › template_SI.pdf]

Supplementary Materials: PiProteline: An R Package for Integrated Proteomics Data Analysis, from Label-Free Quantitation to Systems Biology

Andrea Lomagno [1], Saleh Hamed [1], Ishak Yusuf [1], Pietro Luigi Mauri [1,2] and Dario Di Silvestre [1,*]

1. Benchmarking and comparison

PXD001819 is a public benchmark dataset. A known set of 48 human proteins (called UPS1) was added to a fixed yeast protein background, at nine different known amounts, with three replicates each. The 48 UPS1 proteins should be found as different between two amounts. These are the true positives. The yeast background proteins should not be found as different. If a tool calls them, they are false positives. So we can measure how well each tool actually performs, instead of only counting how many proteins each one reports.

Two comparisons were tested (Figure S1A): P1 vs P4 (a 10-fold difference) and P2 vs P4 (a 5-fold difference). Three replicates per group were considered in both cases. The identity of the 48 UPS1 proteins was checked. All 48 carry a literal "ups" suffix in the data file, and all 48 were cross-checked against the official Sigma list. 48 out of 48 matched. Each tool used its own standard workflow — its own normalization, missing-value handling and statistical test, exactly as its documentation describes. No workflow was adjusted by hand. The significance threshold was made the same for every tool (adjusted $p \leq 0.05$ and $\| \log_2 \text{fold change} \| \geq 1$). This was necessary because the tools use different defaults: limma and protti use only a p-value cutoff, while PiProteline and DEP (Bioconductor) also require a fold-change cutoff. Without this step, we would be comparing thresholds instead of tools. PiProteline was run from the real package code in the repository, not re-written from the description in the manuscript. Normalization was TotSigNorm (default).

Following P1 vs P4 (a 10-fold difference), PiProteline finds 46 of the 48 spike-in proteins, exactly the same as limma. It calls about 6 more background proteins than limma does. The gap between PiProteline, limma and DEP (Bioconductor) is 2 to 6 proteins out of roughly 70 — a small difference (Figure S1B). Similarly, following P2 vs P4 (a 5-fold difference), PiProteline finds 43 of the 48 spike-in proteins, while concerning the previous comparison all tools found much less background proteins Figure S1C); at 10-fold, PiProteline's weakness is extra false positives (27 background proteins called), while at 5-fold, its weakness is misses (5 spike-ins not found, versus 2 or 3 for the others).

Globally, PiProteline is not the best, and we should not claim it is. limma has the best or near-best F1 at both comparisons. PiProteline is third at 10-fold and third at 5-fold. The differences among PiProteline, DEP (Bioconductor) and protti are small and they change between the two comparisons, so we can state it is in the same performance band.

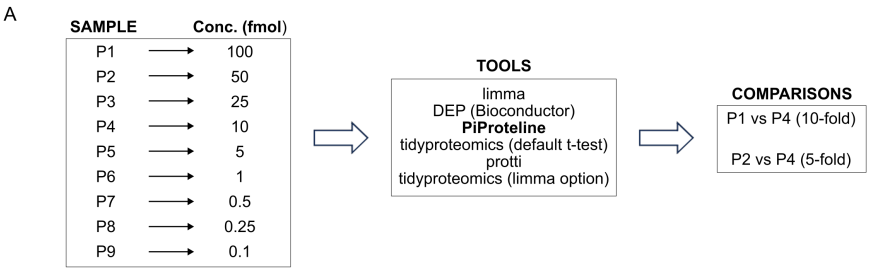

B

Tool	Called	Correct	Wrong	Missed	Sensitivity	FDR	F1
limma	67	46	21	2	0.958	0.313	0.800
DEP (Bioconductor)	70	47	23	1	0.979	0.329	0.797
**PiProteline**	73	46	27	2	0.958	0.370	0.760
tidyproteomics (default t-test)	78	47	31	1	0.979	0.397	0.746
protti	86	47	39	1	0.979	0.453	0.701
tidyproteomics (limma option)	90	47	43	1	0.979	0.478	0.681

C

Tool	Called	Correct	Wrong	Missed	Sensitivity	FDR	F1
tidyproteomics (limma option)	52	46	6	2	0.958	0.115	0.920
limma	50	45	5	3	0.938	0.100	0.918
**PiProteline**	48	43	5	5	0.896	0.104	0.896
protti	55	46	9	2	0.958	0.164	0.893
DEP (Bioconductor)	54	45	9	3	0.938	0.167	0.882
tidyproteomics (default t-test)	1	1	0	47	0.021	0.000	0.041

D

TOOL	DDA/DIA	Quantification	DE statistics	Functional Enrichment	Network Analysis	Other Notable Features
PiProteline	**YES** (output from any search engine)	**YES** (label-free)	MANOVA, Benjamini-Hochberg	**YES**	**YES** (unweighted and weighted network topological analysis for selecting hubs)	R package and Shiny app available on GitHub
aLFQ	**YES** (DDA-LFQ, DIA-LFQ, and plexDDA)	**YES** (IBAQ, TPA values)	Log-Log Linear Regression Monte Carlo Cross-Validation (MCCV)	**NO**	**NO**	Python library (ibaqpy). Scalable for big data
protti	**YES** (output from any search engine)	**YES**	Fixed-Effects Models	**YES**	**YES** (Network analysis using STRING database)	Provides extensive QC functions and direct access to databases like UniProt
mixOmics	**YES** (if already processed)	**NO**	sPCA, sPLS-DA, rCCA, DIABLO, MINT, for dimensionaly reduction	**NO**	**YES** (Relevance Network Graphs, Circos plots for multi-omics)	Designed for data integration and visualization of high-dimensional multi-omics datasets
leapR	**YES** (if already processed)	**NO**	Fisher's exact test, Kolmogorov-Smirnov test, Student's T-Test	**YES**	**YES** (specific for Kinases)	Can calculate enrichment in specified interactions relative to non-pathway members
tidyproteomics	**YES** (data from MaxQuant, PD , FragPipe, Skyline, DIA-NN)	**YES** (label-free, TMT)	Empirical Bayes Student's T-Test	**YES**	**NO**	Focuses on creating a tidy data object for flexible and standardized analysis; supports common data platforms and formats
promor	**YES**	**YES** (label-free)	Linear models and Empirical Bayes	**NO**	**NO**	A comprehensive R package for label-free proteomics data analysis
DEP (Bioconductor)	**YES**	**YES** (label-free, TMT)	Empirical Bayes, Benjamini-Hochberg	**YES**	**NO**	
limma	**YES** (output from any search engine)	**YES** (label-free)	Empirical Bayes	**NO**	**NO**	Often used in proteomics workflows for differential analysis

Figure S1. Comparison of PiProteline and similar tools. A) Comparisons performed (P1 *vs* P4, 10-fold difference; P2 *vs* P4, 5-fold difference) starting from a data matrix obtained by processing a public benchmark dataset (PRIDE ID:PXD001819). **B)** Sensitivity, FDR and F1 values obtained in P1 *vs* P4 and in textbfC) P2 *vs* P4 comparisons. **D)** List of features characterizing PiProteline and a set of similar tools already available; DDA/DIA data, Quantification, Differential Expression (DE) statistics, Functional Enrichment and Network Analysis features were considered.
